# Glucocorticoid receptor and androgen receptor-targeting therapy in patients with castration-resistant prostate cancer

**DOI:** 10.3389/fonc.2022.972572

**Published:** 2022-09-23

**Authors:** Sahyun Pak, Jungyo Suh, Seo Young Park, Yunlim Kim, Yong Mee Cho, Hanjong Ahn

**Affiliations:** ^1^ Department of Urology, Hallym University College of Medicine, Hallym University Kangnam Sacred Heart Hospital, Seoul, South Korea; ^2^ Department of Urology, University of Ulsan College of Medicine, Asan Medical Center, Seoul, South Korea; ^3^ Department of Statistics and Data Science, Korea National Open University, Seoul, South Korea; ^4^ Asan Institute for Life Science, Asan Medical Center, Seoul, South Korea; ^5^ Department of Pathology, University of Ulsan College of Medicine, Asan Medical Center, Seoul, South Korea

**Keywords:** castration-resistant prostate cancer, androgen receptors, glucocorticoid receptors, treatment outcome, survival analysis

## Abstract

**Objective:**

The glucocorticoid receptor (GR) promotes resistance to androgen receptor (AR)-targeting therapies in castration-resistant prostate cancer (CRPC) by bypassing AR blockade. However, the clinical relevance of evaluating GR expression in patients with CRPC has not been determined. The present study investigated the association of relative GR expression in CRPC tissue samples with treatment response to AR-targeting therapy.

**Methods:**

Levels of *GR*, *AR-FL*, and *AR-V7* mRNAs were measured in prostate cancer tissue from prospectively enrolled CRPC patients who were starting treatment. Patients were divided into groups with high and low AR-V7/AR-FL ratios and with high and low GR/AR-FL ratios. The primary endpoint was prostate-specific antigen (PSA) response rate to treatment.

**Results:**

Evaluation of 38 patients treated with AR-targeting therapies showed that the PSA response rate was significantly higher in patients with low than high AR-V7/AR-FL ratios (77.8% vs. 25.0%, p=0.003) and in patients with low than high GR/AR-FL ratios (81.3% vs. 27.3%, p=0.003). Patients with low GR/AR-FL ratios had higher rates of PSA progression-free survival (46.0% vs. 22.4%, p=0.006), radiologic progression-free survival (28.9% vs. 10.0%, p=0.02), and overall survival (75.2% vs. 48.0%, p=0.037) than patients with high GR/AR-FL ratios. The association of GR/AR-FL ratio with PSA response to AR-targeting therapy remained significant in multivariable models. Evaluation of the 14 patients who received taxane chemotherapy showed that PSA response rates did not differ significantly in those with low and high AR-V7/AR-FL and GR/AR-FL ratios, although no definitive conclusions can be drawn due to the small number of patients.

**Conclusion:**

Relative GR expression is associated with sensitivity to AR-targeting therapy and survival in patients with CRPC. Large-scale prospective validation and liquid biopsy-based studies are warranted.

## Introduction

Androgen receptor (AR)-dependent mechanisms are the main pathways for development of castration-resistant prostate cancer (CRPC). Specifically, alternative splicing of *AR* mRNA has emerged as an important mechanism in CRPC progression and resistance to the AR-targeting agents enzalutamide and abiraterone (1). Increased expression of AR splice variants (AR-Vs) is generally accompanied by elevated expression of full-length AR (AR-FL) (2). AR-V7 in particular lacks a ligand-binding domain, but remains transcriptionally active (3). Assessments of AR-V7 mRNA in circulating tumor cells (CTC) (3) showed that the presence of AR-V7 mRNA in CTCs was associated with increased resistance to AR-targeting therapies and that AR-V7 mRNA may be a treatment selection marker in patients with CRPC (4–9).

AR bypass signaling is another mechanism implicated in resistance to AR-targeting therapy. The glucocorticoid receptor (GR), another member of the steroid receptor superfamily of ligand-regulated transcription factors, has structural and functional properties similar to those of AR (10). Importantly, GR contributes to the development of resistance to AR-targeting therapies by bypassing AR blockade in CRPC (11, 12). However, the clinical importance of GR levels in patients with CRPC has not yet been determined. In the present study, we prospectively assessed AR-FL, AR-V7, and GR mRNA expression using tumor tissue from patients with CRPC to investigate the clinical relevance for prediction of treatment response.

## Materials and methods

### Study population

Sixty men with CRPC starting AR-targeting therapy (enzalutamide or abiraterone) or taxane chemotherapy (docetaxel or cabazitaxel) between January 2016 and December 2018 were prospectively enrolled. Men lacking cancerous lesions in the tissue samples (n=4), those not treated after biopsy (n=2), and those from whom mRNA could not be extracted from the biopsy sample (n=2) were excluded. The control group included men with benign prostate hyperplasia (BPH) and those with hormone-naïve prostate cancer. The study protocol was approved by the institutional review board of Asan Medical Center (no. 2014-0957).

### Study design and clinical outcomes

After providing informed consent, all patients underwent transrectal ultrasonography-guided prostate biopsy. Six biopsy cores from each patient were evaluated pathologically to confirm the presence of cancer cells, and two additional cores from target lesions most likely to be cancerous were used in mRNA analysis. AR-FL, AR-V7, and GR mRNA levels were analyzed by real-time quantitative reverse transcription PCR, and AR-FL, AR-V7, and GR protein levels were analyzed by western blotting, as detailed in **Doc S1**.

The choice of treatment was determined by the treating physician, who was blinded to the results of tissue-based mRNA analysis. Follow-up included measurements of serum concentrations of prostate-specific antigen (PSA) levels monthly, and abdominopelvic CT and bone scanning every 3 months. All treatments were continued until PSA progression, radiologic progression, or severe treatment-related adverse events occurred.

The primary endpoint was PSA response rate. The PSA response was defined as a ≥50% reduction from baseline in serum PSA concentration lasting more than 4 weeks. The best PSA response was defined as percent maximal decrease in serum PSA from baseline (3). Secondary endpoints were PSA progression-free survival, radiologic progression-free survival, and overall survival. PSA progression was defined by Prostate Cancer Working Group criteria as a ≥25% increase in serum PSA or more above the nadir to an absolute concentration by ≥2 ng/ml, with confirmation 4 or more weeks later (13). Radiographic progression was determined by CT or bone scans according to Prostate Cancer Working Group criteria (13).

### Statistical analysis

Categorical variables were reported as numbers and frequencies, whereas continuous variables were reported as means and standard deviations (SDs). Correlations between levels of mRNA expression were assessed using Pearson correlation coefficients. PSA response rates between groups were assessed using Fisher’s exact tests. Survival outcomes were evaluated by the Kaplan–Meier method and compared by log-rank tests. Factors significantly associated with survival were assessed by multivariable analysis using the Cox proportional hazard model. To avoid overfitting due to the limited number of patients and events, each multivariable model included only three or four variables. Covariates were selected based on previous findings (3, 4). To evaluate the performance of the fitted model, the areas under the time-dependent receiver operating characteristic (ROC) curves were calculated.

All statistical tests were two-tailed, with a significance level of 0.05. All statistical analyses were performed using R version 3.5.1 software (R Foundation for Statistical Computing, Vienna, Austria).

## Results

### Levels of *AR-FL*, *AR-V7*, *AR-V12*, and *GR* mRNAs in tumor tissue from patients with CRPC

The relative expression of *AR-FL*, *AR-V7*, and *GR* mRNAs in tissue samples from individual patients with CRPC (AR-targeting therapy: n=38; taxane chemotherapy: n=14), BPH (n=2), and hormone-naïve cancer (n=6) is shown in **Figure S1**. *AR-V7* mRNA was detected in 86.5%, and *GR* mRNA was detected in 98.1% of patients with CRPC. Western blot analysis revealed that the levels of AR-FL, AR-V7, and GR proteins were consistent with those of AR-FL, AR-V7, and GR mRNAs (**Figure S2**).

The median AR-V7/AR-FL ratios were 0.9% (IQR, 2.1–16.7%) in hormone-naïve cancers and 1.2% (IQR, 0.2–3.6%) in CRPC, whereas the median GR/AR-FL ratios in these two groups were 35.6% (12.5–128.9%) and 21.8% (IQR, 2.3–72.3%), respectively. *AR-V7* mRNA expression did not correlate significantly with *AR-FL* (r=0.061, p=0.693) or *GR* (r=-0.047, p=0.758) mRNA expression, and *GR* mRNA expression did not correlate significantly with *AR-FL* mRNA expression (r=-0.003, p=0.983).

### Outcomes of patients treated with taxane chemotherapy

Evaluation of the 14 patients who received taxane chemotherapy showed that PSA response rates did not differ significantly in those with low and high AR-V7/AR-FL [60.0% (3/5) vs. 55.6% (5/9), p=1.000] and GR/AR-FL [45.5% (5/11) vs. 100% (3/3), p=0.209] ratios. The best PSA responses in patients treated with taxane chemotherapy, as shown by AR-V7/AR-FL and GR/AR-FL ratios, are shown in **Figure S3**. Kaplan-Meier survival analyses revealed that rates of PSA progression-free survival, radiologic progression-free survival, and overall survival did not differ between those with low and high AR-V7/AR-FL or GR/AR-FL ratios (all log-rank p>0.1). These results suggested that CRPC patients with high GR/AR-FL ratios may be more responsive to taxane than to AR-targeting therapy, although no definitive conclusions can be drawn due to the small number of patients.

### PSA responses in CRPC patients treated with AR-targeting therapy

Of the 38 patients with CRPC starting AR-targeting therapy, including 32 (84.2%) who had not received any prior treatments, 24 (63.2%) were treated with enzalutamide and 14 (36.8%) were treated with abiraterone. The demographic and clinical characteristics of the patients in the CRPC group are summarized in **Table 1**. The median follow-up duration was 26 months.

**Table 1:** Baseline demographic and clinical characteristics of patients with CRPC starting AR-targeting therapy.

	Total number of patients N=38
Age (years, median)	69.5
ECOG performance status	
0	20 (52.6)
1–2	18 (47.4)
ADT duration (median, months)	28
Prior CRPC treatments	
None	32 (84.2)
AR-targeting therapy	1 (2.6)
Chemotherapy	5 (13.2)
M stage	
M0	4 (10.5)
M1a	2 (5.3)
M1b	31 (81.6)
M1c	1 (2.6)
Bone metastasis	
None	6 (15.8)
1–3 metastatic lesions	16 (42.1)
>3 metastatic lesions	16 (42.1)
Baseline laboratory findings (median)	
Prostate-specific antigen (ng/ml)	20.0
Hemoglobin (median, g/dl)	12.6
Platelet count (median)	197,500
Albumin (median, g/dl)	3.9
Alkaline phosphatase (median, U/L)	91.5
Lactate dehydrogenase (median, U/L)	192.5

**Figure 1:**
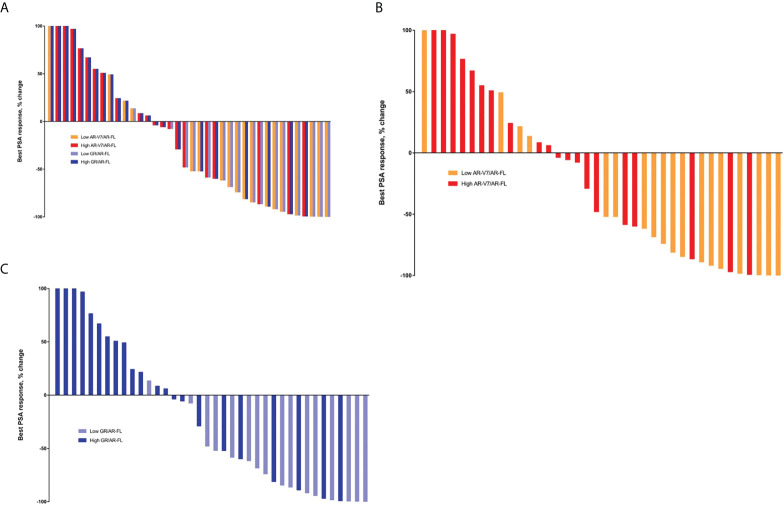
Best PSA responses in patients treated with AR-targeting therapy. **(A)** Waterfall plot based on both AR-V7/AR-FL and GR/AR-FL ratios. **(B)** Waterfall plot based on AR-V7/AR-FL ratios. **(C)** Waterfall plot based on GR/AR-FL ratios.

The overall PSA response rate in patients treated with enzalutamide or abiraterone was 50.0% (19 of 38). Analysis of patients receiving AR-targeting therapy showed that the PSA response rate was significantly higher in patients with low than high AR-V7/AR-FL ratios (77.8% vs. 25.0%, p=0.003; **Table 2**), as well as being significantly higher in patients with low than high GR/AR-FL ratios (81.3% vs. 27.3%, p=0.003). No differences were observed between patients treated with enzalutamide and abiraterone (**Table S1**). PSA response rates were 91.7% (11/12) in patients with both low GR/AR-FL and low AR-V7/AR-FL ratios, and 18.8% (3/16) in patients with both high GR/AR-FL and high AR-V7/AR-FL ratios. ROC analysis showed that the areas under the curve (AUC) were 0.773 for AR-V7/AR-FL ratios and 0.770 for GR/AR-FL ratios (**Figure S4**). The best PSA responses in patients treated with AR-targeting agents, as determined by their AR-V7/AR-FL and GR/AR-FL ratios, are shown in **Figure 1**. Logistic regression models (**Table S2**) showed that both high GR/AR-FL ratio [hazard ratio (HR), 0.142; 95% confidence interval (CI), 0.022–0.756; p=0.027] and high AR-V7/AR-FL ratio (HR, 0.167; 95% CI, 0.029–0.848; p=0.034) independently predicted PSA response.

**Table 2:** PSA response rates in patients with CRPC treated with AR-targeting agents in relation to AR-V7/AR-FL and GR/AR-FL ratios.

		GR/AR-FL		
		Low	High	Total
AR-V7/AR-FL	Low	91.7% (11/12)	50.0% (3/6)	77.8% (14/18)
	High	50.0% (2/4)	18.8% (3/16)	25.0% (5/20)
	Total	81.3% (13/16)	27.3% (6/22)	

### Survival outcomes in CRPC patients treated with AR-targeting therapy

**Figure 2:**
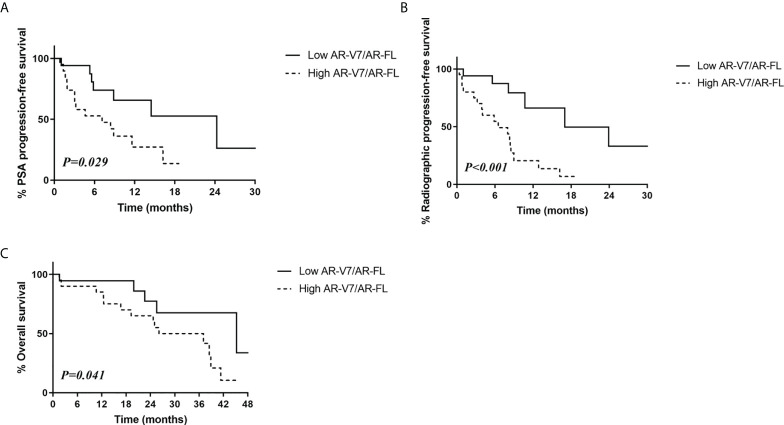
Relationship between survival outcomes and AR-V7/AR-FL ratios in CRPC patients treated with AR-targeting therapy. **(A)** PSA progression-free survival. **(B)** Radiologic progression-free survival. **(C)** Overall survival.

Evaluation of patients treated with AR-targeting therapy showed that those with low AR-V7/AR-FL ratios had significantly higher PSA progression-free survival (52.6% vs. 13.6%, p=0.029; **Figure 2A**) and radiologic progression-free survival (33.1% vs. 6.8%, p<0.001; **Figure 2B**) rates than patients with high AR-V7/AR-FL ratios. The 3-year overall survival rate was significantly higher in patients with low AR-V7/AR-FL than high AR-V7/AR-FL ratios (67.6% vs. 50.0%, p=0.041; **Figure 2C**).

**Figure 3:**
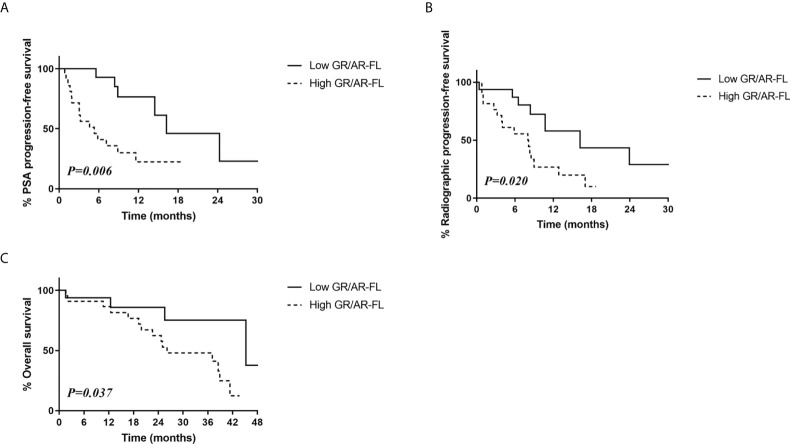
Relationship between survival outcomes and GR/AR-FL ratios in CRPC patients treated with AR-targeting therapy. **(A)** PSA progression-free survival. **(B)** Radiologic progression-free survival. **(C)** Overall survival.

Similarly, patients with low GR/AR-FL ratios had significantly higher PSA progression-free survival (46.0% vs. 22.4%, p=0.006; **Figure 3A**) and radiologic progression-free survival (28.9% vs. 10.0%, p=0.02; **Figure 3B**) rates than patients with high GR/AR-FL ratios. The 3-year overall survival rate was also significantly higher in patients with low GR/AR-FL than high GR/AR-FL ratios (75.2% vs. 48.0%, p=0.370; **Figure 3C**).

## Discussion

Although new therapeutic agents have improved survival in CRPC patients, a substantial number of patients do not benefit from first- or second-line treatments. Additionally, the mechanisms of cross-resistance between therapies and optimal treatment sequencing are not fully understood (14). These challenges highlight the importance of biomarkers to predict treatment response.

Most studies investigating the clinical implications of expression of AR-FL and AR-Vs in CRPC have reported that CTC-based detection of AR-V7 predicts nonresponse to AR-targeting therapies (3, 6–9). More importantly, detection of AR-V7 in CTCs from patients with CRPC was found to correlate with better survival outcomes in response to taxane chemotherapy than to AR-targeted therapy, suggesting that CTC AR-V7 may act as a treatment selection marker (4, 6, 8). Other studies, however, have reported that AR-V7 status in CTCs cannot fully predict nonresponse to AR-targeting therapies (15, 16). These findings suggest the need to identify additional biomarkers that can predict responses to treatment.

The present study confirmed that increased AR-V7 expression relative to AR-FL is associated with resistance to AR-targeting agents. In agreement with previous studies, patients with high AR-V7/AR-FL ratios were found to have poorer responses to treatment with enzalutamide and abiraterone and poorer survival outcomes than patients with low AR-V7/AR-FL ratios (3, 4, 7). The present study analyzed the effects of tumor AR-V7/AR-FL ratios rather than AR-V7 detection in CTCs on patient outcomes, which may contribute to difference in significant factors between studies. AR-V7 mRNA was detected in 86.5% of CRPC tissue biopsies, compared with 24–46% of samples in previous CTC-based studies. Conversely, the median AR-V7/AR-FL mRNA ratio was 1.2%, markedly lower than in CTC-based studies. These disparate results may be due to differences in methodologies, as most studies evaluating the relevance of AR-V7 in CRPC were based on CTC-positive blood samples (3–9, 15), utilizing different target specimens and methods for detecting AR-V7 (2, 17–22). CTCs are thought to represent cells or cellular material derived from metastatic tumors (14). However, many patients with CRPC do not have detectable blood CTCs, and failure to detect CTCs is not indicative of AR-V7 negative disease (23).

AR-FL and AR-V7 are generally co-expressed, and the clinical significance of relative expression ratios has been investigated. AR-V7/AR-FL ratios have been found to vary significantly among detection methods and target specimens. In most CTC-based studies, the median AR-V7/AR-FL ratio was >20% (3, 4, 7) but it was <5% in some tissue-based analyses (17, 20), consistent with the present findings. In the present study, the median AR-V7/AR-FL and GR/AR-FL ratios of patients with hormone-naïve cancer were regarded as cutoff values for subgroup analysis. Ratios in hormone-naïve patients rather than ratios in the study sample were used as cutoffs to avoid false positives due to a data-driven approach. However, use of cutoffs based on median ratios in CRPC tissues yielded similar results (**Figure S5**).

**Figure 4:**
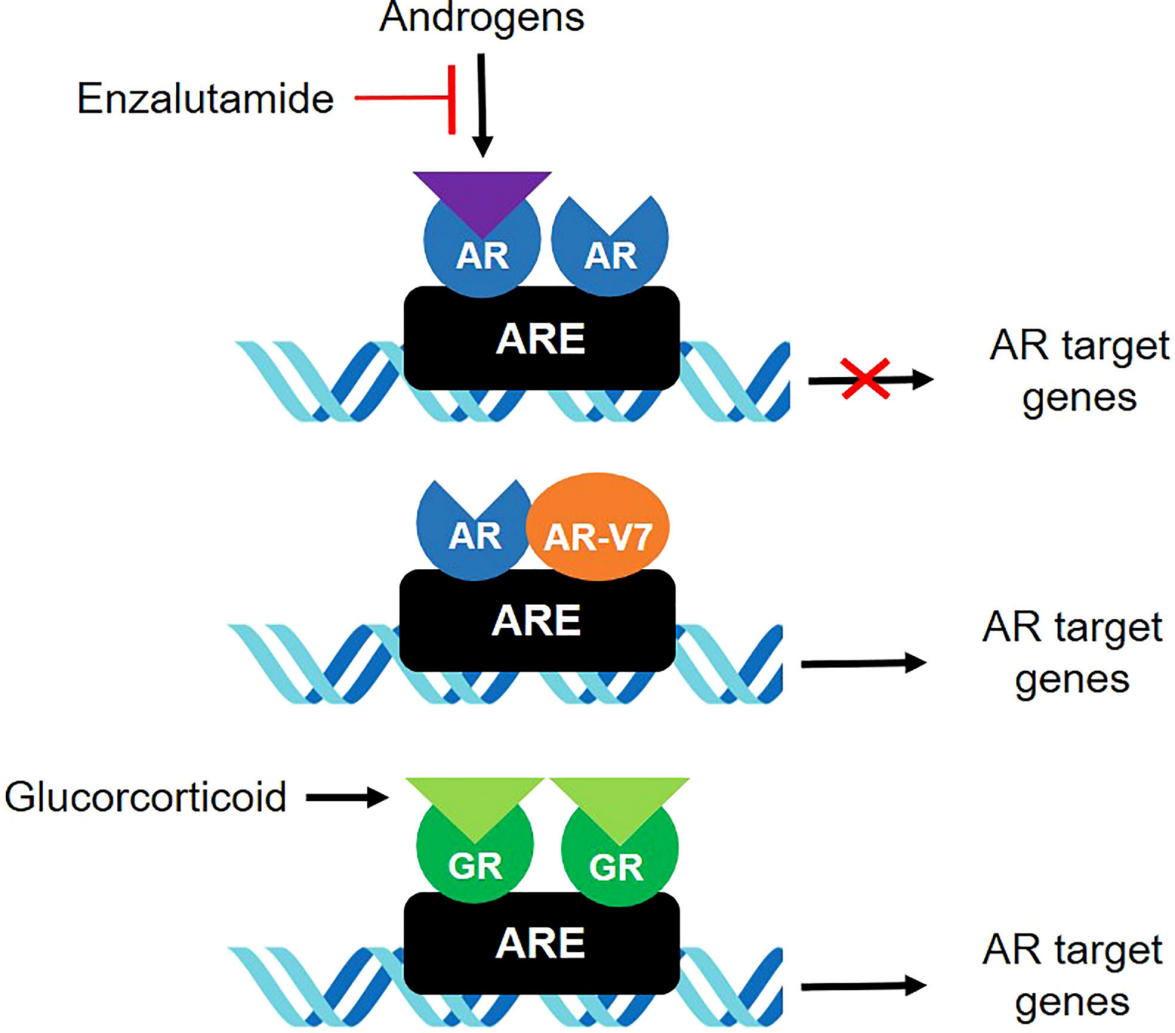
GR and AR-V7 in androgen-independent mechanisms involved in resistance of CRPC to treatment.

The present study focused on GR expression in patients with CRPC. Glucocorticoids have long been taken into account for management of patients with CRPC (24). Several studies investigated the role of GR-regulated escape mechanisms in AR pathway resistance in CRPC (11, 12, 25, 26). GR upregulation has been associated with acquired resistance to AR signaling inhibitors (**Figure 4**), with relatively low levels of endogenous GR being sufficient to confer resistance to androgen-targeting agents in CRPC cells (11). In addition, analysis of AR and GR mRNAs revealed that these two receptors had highly overlapping transcriptome and cistrome profiles (11, 25).

GR-mediated bypass may occur during early stages of CRPC development (1). GR is elevated in docetaxel-resistant cell lines and bone metastases of prostate cancer patients after enzalutamide therapy (26). Although abiraterone acetate, a potent androgen synthesis inhibitor, may not be directly associated with GR signaling, significant amount of adrenal androgen precursors, such as dehydroepiandrosterone-sulfate, have been found to persist after abiraterone treatment, which may lead to the intratumoral biosynthesis of dihydrotestosterone (DHT) (27, 28). DHT enhances the proliferation of CRPC cells devoid of AR through GR pathways involving signal transducer and activator of transcription 5 (STAT-5) activation (29). These findings suggested that increased GR expression relative to AR may be associated with reduced treatment response to enzalutamide and abiraterone. Indeed, the present study found that PSA response was poorer in patients with high than low GR/AR-FL ratios, and that higher GR/AR-FL ratios were associated with poorer PSA progression-free, radiologic progression-free, and overall survival. Further, GR assessment may be a better predictor of response to treatment when combined with AR-V7 assessment. These results suggest that increased GR expression relative to AR-FL may be associated with poor response to AR-targeting agents and survival in patients with CRPC, and that relative GR expression may predict response to AR-targeting agents, either alone or in combination with AR-V7 expression. Although evidence to support a direct relationship between AR-V7 and GR is limited, AR-V7 interacts with ligand-bound members of the NR3C-family of nuclear hormone receptors, including full-length AR and GR. One study showed AR-V7 and GR could occupy the same genomic sites in a ligand-dependent manner, suggesting that AR-V7 can utilize GR as an alternative binding partner to initiate heterodimerization transcriptional activation (30).

These findings also support the use of GR antagonists for CRPC (31, 32). Knockdown or pharmacologic inhibition of GR has been found to reduce the proliferation of prostate cancer cells (26, 29). Although GR inhibition may be ineffective and potentially harmful (24, 33), ongoing clinical trials (NCT02012296 and NCT03437941) are evaluating the effects of GR antagonist combined with enzalutamide in patients with CRPC. The results of these trials may determine whether GR inhibition has clinical efficacy in patients with CRPC.

The present study has several limitations. First, the patient cohort was small, and the absence of an independent validation set precluded drawing definitive conclusions from the results of this prospective study. Moreover, the number of patients who received taxane chemotherapy was too small to examine the association of relative AR and GR expression with taxane sensitivity. Second, prostate biopsy specimens may not represent the overall tumor burden due to potential tumor heterogeneity. In addition, cancer cells were not observed in biopsy specimens from four patients (6.7%), who were excluded from analysis. The tissue biopsy approach also has the advantage of a higher *AR-V7* mRNA detection rate compared with CTC-based studies, thus enabling more accurate quantification. If a CTC-based study is not possible, tissue-based analysis based on prostate biopsies may be useful for assessing molecular biomarkers in selected patients. However, this strategy might not be suitable for clinical practice settings, as target lesions for biopsy are not always present, and prostate needle biopsy is an invasive procedure. Therefore, future studies should be performed with non-invasive methods using sampling approaches such as CTCs or cell-free nucleic acids from blood or urine.

In summary, the present findings support an association between relative GR expression and resistance to AR-targeting therapy and survival in patients with CRPC. Large-scale prospective validation and liquid biopsy-based studies are warranted.

## Data availability statement

The original contributions presented in the study are included in the article/**Supplementary Materials**. Further inquiries can be directed to the corresponding author.

## Ethics statement

The study protocol was approved by the institutional review board of Asan Medical Center (no. 2014-0957). The patients/participants provided their written informed consent to participate in this study.

## Author contributions

SP and HA designed the study, performed the data analysis, and drafted the manuscript. SYP performed statistical analysis. SP, JS, YK, YC, HA participated in the data acquisition. HA supervised the project. All authors discussed the results and commented on the manuscript. All authors contributed to the article and approved the submitted version.

## Funding

This study was supported by a grant (W17-012) from the Asan Institute for Life Sciences, Asan Medical Center, Seoul, Korea.

## Conflict of interest

The authors declare that the research was conducted in the absence of any commercial or financial relationships that could be construed as a potential conflict of interest.

## Publisher’s note

All claims expressed in this article are solely those of the authors and do not necessarily represent those of their affiliated organizations, or those of the publisher, the editors and the reviewers. Any product that may be evaluated in this article, or claim that may be made by its manufacturer, is not guaranteed or endorsed by the publisher.

## Supplementary material

Doc S1 Methods

Supplementary Figure 1 Relative expression of AR-FL, AR-V7, and GR mRNAs in each patient.

Supplementary Figure 2 Western blot analysis of AR-FL, AR-V7, and GR protein levels in patient tissue samples.

Supplementary Figure 3 Best PSA responses in patients treated with taxane chemotherapy.

Supplementary Figure 4 Receiver operating characteristic curve analysis for prediction of PSA response.

Supplementary Figure 5 Outcomes in patients treated with AR-targeting therapy using median AR-V7/AR-FL and GR/AR-FL ratios in CRPC tissue samples as cutoff points. **(A)** Best PSA responses. **(B)** PSA progression-free survival according to AR-V7/AR-FL. **(C)** PSA progression-free survival according to GR/AR-FL.

Supplementary Table 1 PSA responses in patients treated with enzalutamide or abiraterone.

Supplementary Table 2 Logistic regression analyses predicting PSA response in patients treated with AR-targeting therapy.
