# Supplementary material for: Glucocorticoid receptor and androgen receptor-targeting therapy in patients with castration-resistant prostate cancer
Source: Front Oncol. 2022 Sep 23;12:972572. doi: 10.3389/fonc.2022.972572 (PMC9541428; doi:10.3389/fonc.2022.972572)
Supplement: Doc S1 — Methods [file DataSheet_1.docx]

**Doc. S1. Methods**

**Prostate cancer specimens and RNA extraction**

Prostate cancer tissues were obtained from patients who underwent a transrectal ultrasonography-guided needle biopsy and were subsequently diagnosed with prostate cancer at Asan Medical Center (AMC). The study protocol was approved by the Institutional Review Board (IRB) of AMC (no. 2014-0957), and all patients provided written informed consent. Frozen specimens were ground to powder form in liquid nitrogen using a mortar and pestle, and total RNA was extracted with Trizol^®^ reagent (Thermo Fisher Scientific, Waltham, MA, USA), according to the manufacturer’s instructions.

**Real-time quantitative reverse transcription PCR**

Total RNA (2 μg) samples were reverse transcribed to cDNA using cDNA Synthesis Kits (Toyobo, Osaka, Japan). Specific sequences in these cDNA samples were amplified by real-time PCR using an ABI 7500 Fast sequence detector system (Applied Biosystems, Foster City, CA, USA) and specific primers for AR (forward, 5’-CAGTGGATGGGCTGAAAAAT-3’; reverse, 5’-AAGCGTCTTGAGCAGGATGT-3’); GR (forward, 5’-GCGATGGTCTCAGAAACCAAAC-3’; reverse, 5’-GCAGAGGATAACTTCCTCTGTAATCTC-3’); AR-V7 (forward, 5’-CAGGGATGACTCTGGGAGAA-3’; reverse, 5’-GCCCTCTAGAGCCCTCATTT-3’); AR-V12 (forward, 5’-GCCATTGAGCCAGGTGTAGT-3’; reverse, 5’-TGCAGCTCTCTCGCAATAGG-3’); and GAPDH (forward, 5’-CAATGACCCCTTCATTGACC-3’; reverse, 5’-GACAAGCTTCCCGTTCTCAG-3’). The amplification protocol consisted of an initial denaturation at 95°C for 20 seconds, followed by 40 cycles of denaturation at 95°C for 3 seconds, and annealing and extension at 60°C for 30 seconds. Melting curves were determined by denaturation at 95°C for 15 seconds, melting at 60°C for 1 minute increasing to 95°C for 15 seconds at a ramp rate of 1%, and 60°C for 15 seconds. Relative expression was calculated by dividing C_T_ values of *AR-FL*, *AR-V7*, and *GR* mRNAs by the C_T_ value of *GAPDH* mRNA in the same sample. PCR amplification efficiency and linearity were tested for each gene, including target and control genes.

**Western blot analysis**

Cells were lysed in lysis buffer [150 mmol/L NaCl, 1% Nonidet P-40, 50 mmol/L Tris-HCl (pH 7.4), 50 mmol/L NaF, 5 mmol/L EDTA, 0.1 mmol/L Na_3_VO_4_, and 0.1% SDS] containing protease inhibitor (Sigma Aldrich, St Louis, MO, USA). The cell lysates were centrifuged at 13,000 × g for 10 minutes, and supernatants were stored at 4°C. Protein sample concentrations were measured using a Bradford protein assay (Bio-Rad, Hercules, CA, USA). Equal amounts of proteins were subjected to sodium dodecyl sulfate (SDS)-polyacrylamide gel electrophoresis (PAGE), and transferred to PVDF membrane. The membranes were stained with Ponceau red to confirm equal sample loading and transfer. After blocking with 5% bovine serum albumin (BSA) for 1 hour at room temperature, the membranes were incubated overnight at 4°C with primary antibodies while shaking at 100 rpm. The membranes were washed and incubated with peroxidase-conjugated secondary antibodies for 1 hour at RT. Immunoreactive bands were visualized on X-ray film using Immobilon Western enhanced chemiluminescent (ECL) solution (Millipore Corp., Burlington, MA, USA). β-actin was used as an internal loading control.

**Statistical analysis**

Because the distributions of GR/AR-FL and AR-V7/AR-FL ratios were both highly skewed to the right, the ratios were log-transformed to achieve approximate normality. The ratios were dichotomized, as the associations between ratios and outcomes may not have been linear. Median ratios of hormone-naïve patients were used as cutoffs rather than median ratios of the study samples to avoid increasing the likelihood of false positives due to the use of a data-driven approach. To analyze log values, the PCR-determined copy number of tissues with undetectable mRNA was set at 10^-5^.

Sample size was based on the primary endpoint, PSA response. It was hypothesized that the proportions of patients that could be dichotomized according to GR expression and PSA response rates would be ≤ 25% in patients with highly expressed GR and ≥70% in patients with little or no GR expression. Based on this assumption, a sample size of 38 patients was calculated to give the study 80% power to detect a difference of 45% in PSA response rates.

**Figure S1. Relative expression of *AR-FL*, *AR-V7*, and *GR* mRNAs in each patient.**


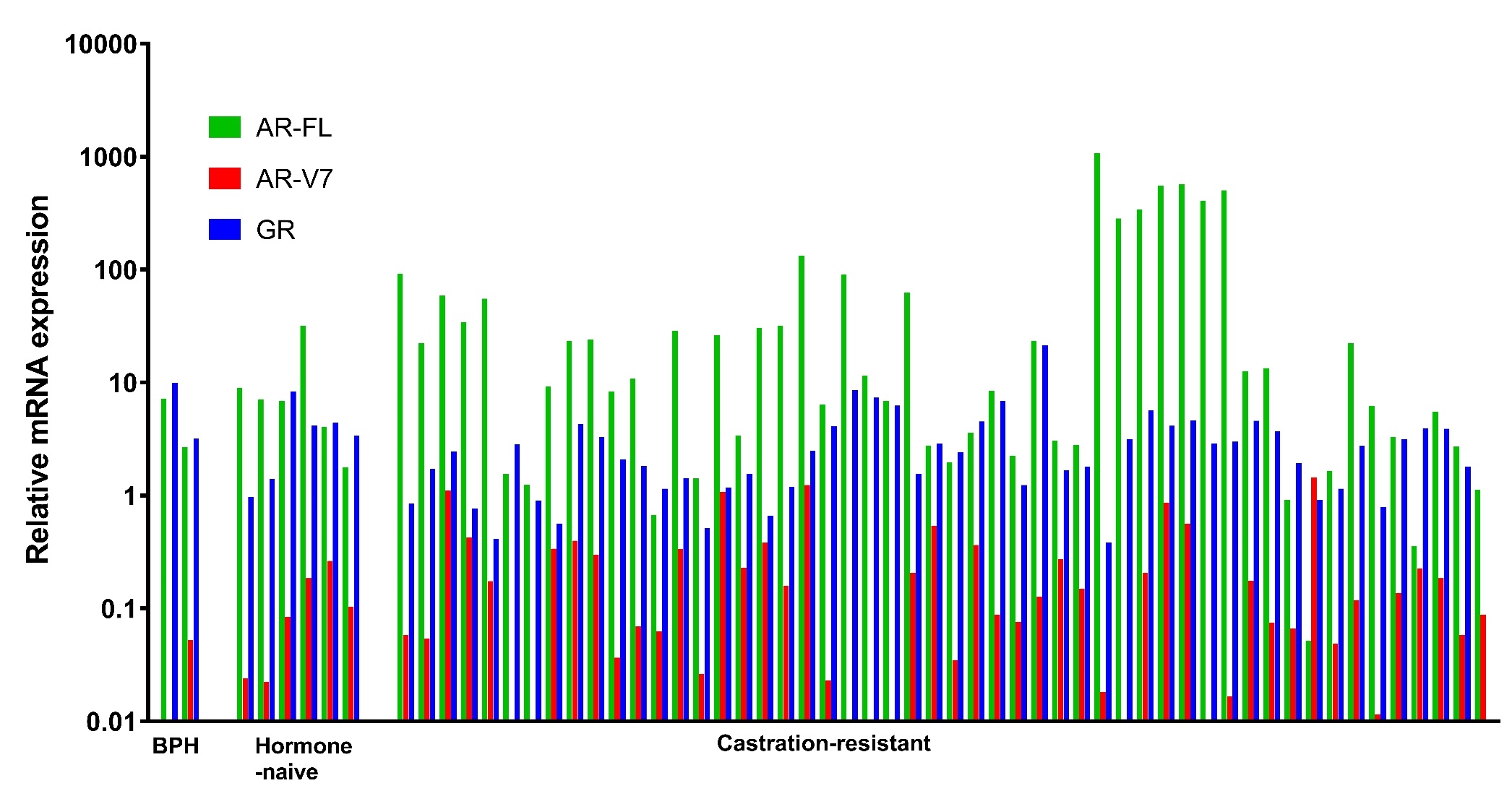


**Figure S2. Western blot analysis of AR-FL, AR-V7, and GR protein levels in patient tissue samples. CRPC#1 sample is derived from patient who had not received any prior treatments, and CRPC#2 is from patient who progressed after docetaxel treatment.**


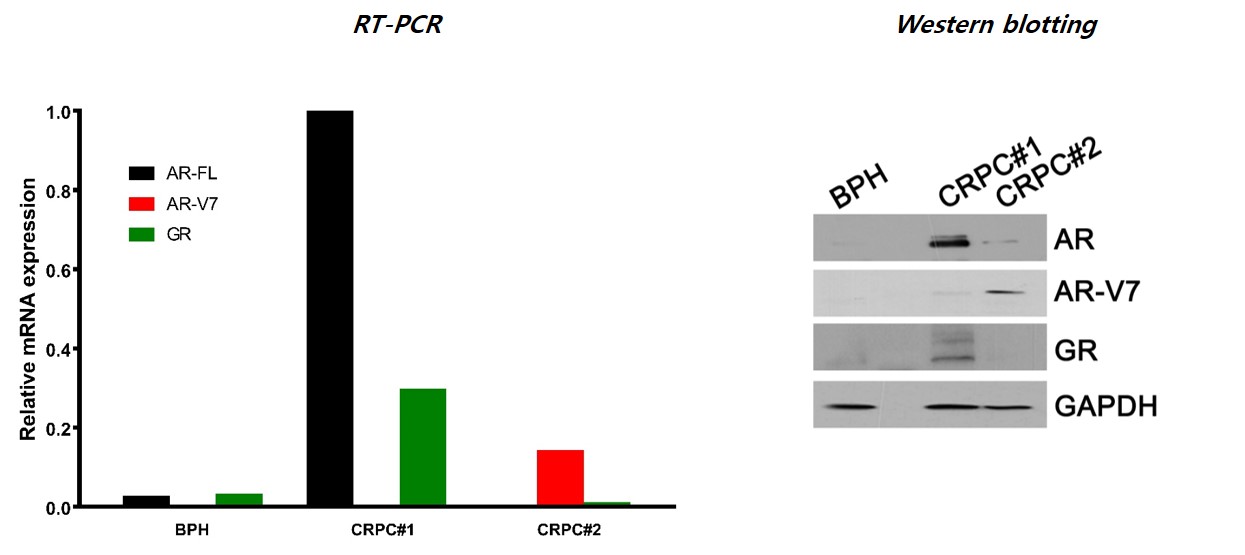


**Figure S3. Best PSA responses in patients treated with taxane chemotherapy.**


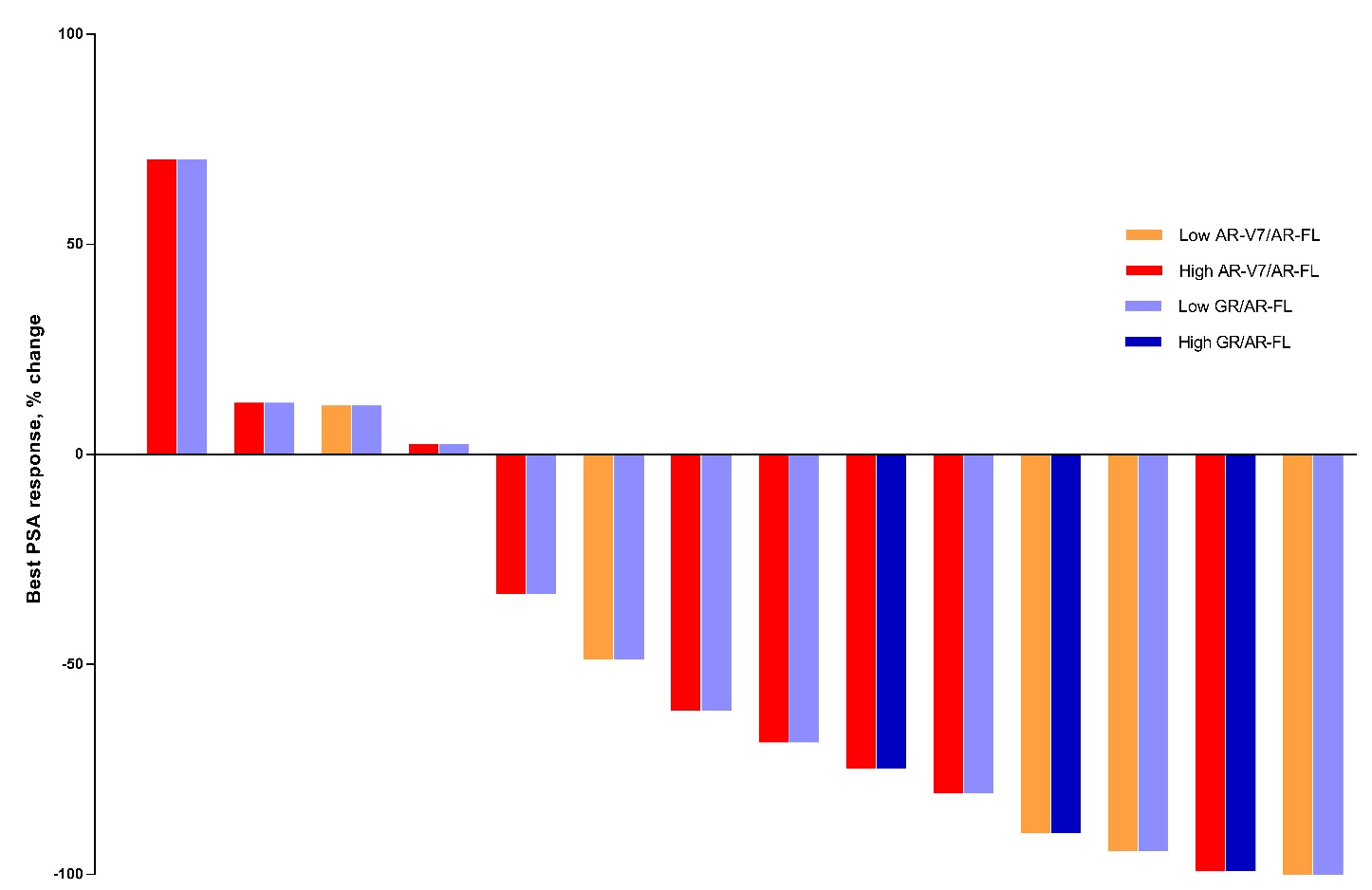


**Figure S4. Receiver operating characteristic curve analysis for prediction of PSA response.**


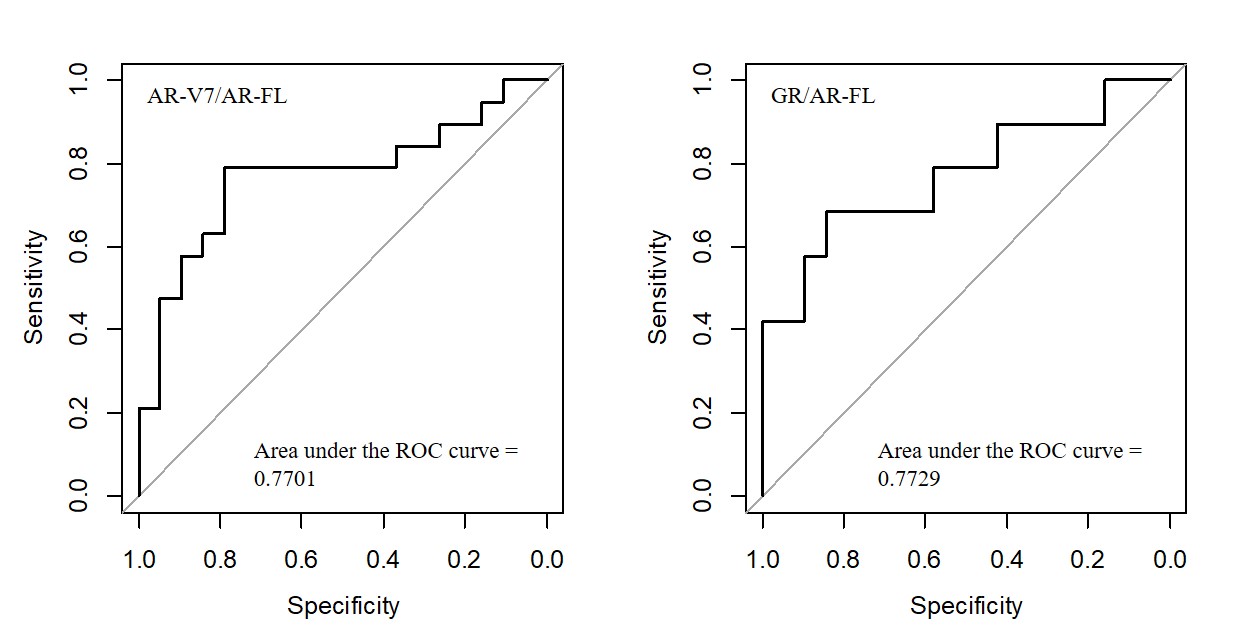


**Figure S5. Outcomes in patients treated with AR-targeting therapy using median AR-V7/AR-FL and GR/AR-FL ratios in CRPC tissue samples as cutoff points.**

(A) Best PSA responses.

(B) PSA progression-free survival according to AR-V7/AR-FL.

(C) PSA progression-free survival according to GR/AR-FL.


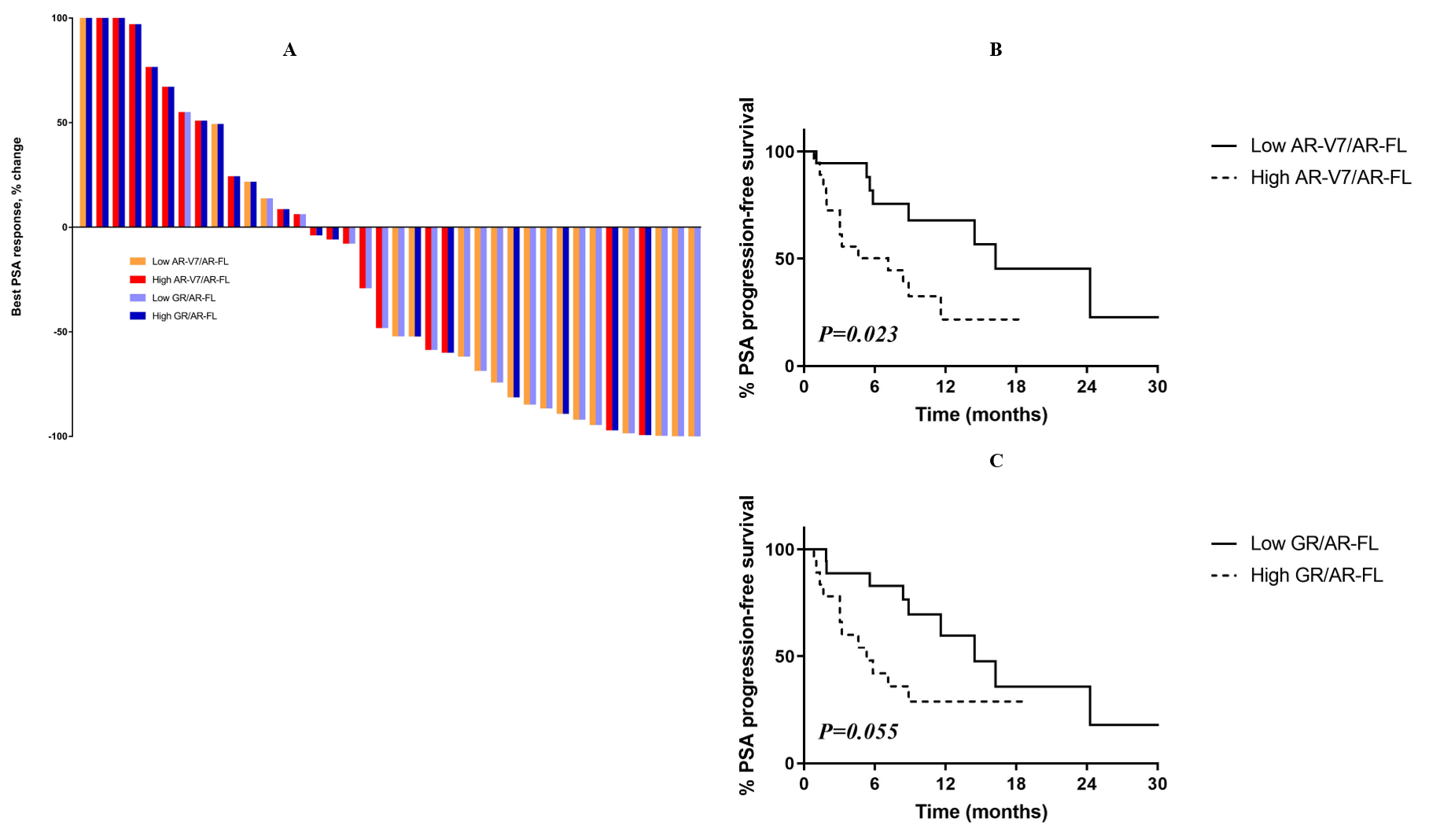


**Table S1. PSA responses in patients treated with enzalutamide or abiraterone.**

|  | PSA response (%) | | | |
| --- | --- | --- | --- | --- |
| Treatment | High AR-V7/AR-FL | Low AR-V7/AR-FL | High GR/AR-FL | Low GR/AR-FL |
| Enzalutamide | 5/16 (31.3) | 6/8 (75.0) | 5/15 (33.3) | 6/9 (66.7) |
| Abiraterone | 0/4 (0.0) | 8/10 (80.0) | 1/7 (14.3) | 7/7 (100.0) |

**Table S2. Logistic regression analyses predicting PSA response in patients treated with AR-targeting therapy.**

|  | Univariate | | | | Multivariable (full model, continuous ratios) | | | | Multivariable (full model, dichotomous ratios) | | | |
| --- | --- | --- | --- | --- | --- | --- | --- | --- | --- | --- | --- | --- |
|  | OR point est. | OR 95% CI | | p-value | OR point est. | OR 95% CI | | p-value | OR point est. | OR 95% CI | | p-value |
|  |  | LB | UB |  |  | LB | UB |  |  | LB | UB |  |
| GR/AR-FL (dichotomous) | 0.087 | 0.015 | 0.374 | 0.002 |  |  |  |  | 0.142 | 0.021 | 0.778 | 0.030 |
| AR-V7/AR-FL (dichotomous) | 0.095 | 0.019 | 0.394 | 0.002 |  |  |  |  | 0.167 | 0.028 | 0.862 | 0.036 |
| GR/AR-FL (continuous) | 0.504 | 0.280 | 0.771 | 0.006 | 0.553 | 0.296 | 0.881 | 0.027 |  |  |  |  |
| AR-V7/AR-FL (continuous) | 0.621 | 0.398 | 0.853 | 0.013 | 0.670 | 0.416 | 0.985 | 0.063 |  |  |  |  |
| Baseline PSA | 0.922 | 0.613 | 1.358 | 0.679 | 0.876 | 0.523 | 1.421 | 0.587 | 0.885 | 0.534 | 1.402 | 0.605 |
| Treatment sequence | 1 | 0.202 | 4.958 | >0.999 | 0.405 | 0.034 | 3.293 | 0.419 | 1.007 | 0.125 | 7.638 | 0.994 |

|  | With ratios as continuous vars | With ratios as dichotomous vars |
| --- | --- | --- |
| AUCs |  |  |
| Apparent AUC | 0.856 | 0.834 |
| Optimism-corrected AUC | 0.793 | 0.769 |
